# Sounding Black or White: priming identity and biracial speech

**DOI:** 10.3389/fpsyg.2015.00457

**Published:** 2015-04-20

**Authors:** Sarah E. Gaither, Ariel M. Cohen-Goldberg, Calvin L. Gidney, Keith B. Maddox

**Affiliations:** ^1^Department of Psychology, University of Chicago, Chicago, ILUSA; ^2^Center for the Study of Race, Politics, and Culture, University of Chicago, Chicago, ILUSA; ^3^Department of Psychology, Tufts University, Medford, MAUSA; ^4^Eliot-Pearson Department of Child Study and Human Development, Tufts University, Medford, MAUSA

**Keywords:** biracial identity, priming, language, speech perceptions, styleswitching

## Abstract

Research has shown that priming one’s racial identity can alter a biracial individuals’ social behavior, but can such priming also influence their speech? Language is often used as a marker of one’s social group membership and studies have shown that social context can affect the style of language that a person chooses to use, but this work has yet to be extended to the biracial population. Audio clips were extracted from a previous study involving biracial Black/White participants who had either their Black or White racial identity primed. Condition-blind coders rated Black-primed biracial participants as sounding significantly more Black and White-primed biracial participants as sounding significantly more White, both when listening to whole (Study 1a) and thin-sliced (Study 1b) clips. Further linguistic analyses (Studies 2a–c) were inconclusive regarding the features that differed between the two groups. Future directions regarding the need to investigate the intersections between social identity priming and language behavior with a biracial lens are discussed.

## Introduction

People have multiple social identities based on group memberships, social roles, and affiliations that can become more or less salient over time and across context (i.e., race, gender, age, occupation). This kind of social identity priming can be understood from the perspective of social identity theory (Tajfel and Turner, 1986) which states that an individual’s self-concept is defined based on one’s perceived group membership. Moreover, one’s social identity has been proven to be an important source of self-esteem, behavior, one’s sense of belonging, and purpose in the social world (Tajfel and Turner, 1986; Correll and Park, 2005). Social identity priming reveals that the salience of various identities can easily be swayed by cues in the environment. For example, we may identify more with our occupation when at work, but when at home other aspects of our identities (e.g., as parents or spouses) may become more salient. Similarly, chronic or momentary cues to our racial, ethnic, gender, or occupational identity may also subtly influence our behavior.

Research has explored a variety of contexts that can prime one’s social identity. However, there is a particularly understudied population within the social identity theory framework—biracial individuals (those with parents from two different racial backgrounds). Recent research has highlighted the fact that that social context can significantly alter how biracial individuals racially identify, forcing them to navigate between their different racial identities subconsciously (e.g., Chiao et al., 2006; Cheng and Lee, 2009; for a review see Gaither, 2015). More specifically, a simple racial identity priming task has been shown to affect how much biracial Black/White individuals identify and socially interact with other Black or White people (Gaither et al., 2013). Therefore it is clear that racial identity is a psychological mechanism that elicits changes on biracial individuals’ behavior. We know that a biracial person’s identification is influenced by a number of contextual and interpersonal variables including the racial group membership of one’s interaction partner. But what remains unknown is whether this shift in identity caused by racial priming can also shape other aspects of the way biracial individuals express their identity such as their verbal behavior independent of the race of their interaction partner.

In fact, language is one of the most prominent means of expressing one’s social identity. Previous work has shown that context can influence one’s language use, suggesting a degree of malleability similar to other manifestations of identity (e.g., Giles and Johnson, 1981, 1987; Gumperz, 1982; Ochs, 1993; Pennebaker et al., 2003). Unfortunately, while it is clear that language use is influenced by one’s social context, the specific mechanisms by which social identity influences language use in the moment are not clear. This is partly a result of the fact that research in the social psychological and sociolinguistic domains has typically proceeded independently of each other. Generally speaking, social psychologists probe the social and cognitive factors involved in the formation and expression of identity while sociolinguists investigate the systematic ways that language use varies between different social groups and contexts. Unfortunately, very little interaction exists between these literatures, despite the fact that identity strongly manifests itself through language use, which can substantially vary based on social affiliation. The goal of the present investigation was to begin to bridge these disciplines and explore the specific cognitive mechanisms that support the connection between social identity and language. To do so, we focused on the speech of a group that regularly moves between different social identities—the biracial Black/White population (e.g., Chiao et al., 2006; Cheng and Lee, 2009; Gaither et al., 2013; Gaither, 2015).

Both styleswitching and codeswitching are defined as a moment when people alter their speech between one or more speaking styles. While the terms are often used interchangeably in the literature, they differ subtly. *Styleswitching* usually refers to an intentional stylistic switch in speaking to align with one’s context or with one’s perceived identity in a given situation whereas *codeswitching* often occurs to resolve basic communicative needs such as when a bilingual individual only knows the name of an object in one of their languages (e.g., Labov, 1972). Therefore, styleswitching most commonly occurs in response to one’s audience and the topic at hand and may involve all levels of linguistic structure, with shifts in syntactic, morphological, and phonological patterns as well as word choice and low-level phonetic features (Blom and Gumperz, 1972; Gumperz, 1982). One early theory, proposed by Labov (1996), is that speech style varies in relation to the amount of attention paid by a speaker to his or her speech. According to Labov (1996), speech may be generally seen to span a continuum from ‘casual’ (the speech used in everyday situations when no attention is being paid to how one is speaking) to ‘careful’ (e.g., the speech used when one knows he or she is being recorded), with linguistic features associated with formality appearing in the latter but not the former. A more comprehensive theory was proposed by Bell (1984), who argued that styleswitching is primarily a form of *audience design*, where speakers shape their speech directly in response to the identity of their interlocutors. On this view, styleswitching is often a form of accommodation whose purpose is to create (or in some cases, reduce) ‘alignment’ among interlocutors (e.g., Fuller, 1993; Bucholtz, 2009).

In fact, to date, sociolinguistic theorizing has tended to make the external factors their object of focus. The audience design proposal, for example, locates one’s addressees as the primary determinants of styleswitching (e.g., Giles and Powesland, 1975; Gumperz, 1982; Bullock and Toribio, 2009). Speakers are always crafting (consciously or unconsciously) their speech in relation to the social identity of one’s interlocutor. Styleswitching is also an important means of expression for individuals who are navigating multiple social or cultural identities. Benor (2012), for example, reports that Baalei Teshuva (Jews who are becoming more observant) styleswitch between Jewish dialects of English as part of their “hybrid self-presentation.” This highlights how important styleswitching is for the expression and management of identity as well as how easily bicultural individuals can accommodate their speaking styles situationally based on their current sense of self or social identity. In this vein, sociolinguistic research has tended to investigate styleswitching by manipulating the factors external to a speaker such as different interlocutors, environments, topics, and so on.

In the present study, we take this approach in a new direction by manipulating speakers’ self-concept and observing the effects on speech. This approach allows us to ask two primary questions. First, the identity priming techniques developed by social psychologists have been shown to influence relatively high-level social processes such as behavioral tendencies and social identification; we ask whether it can also influence relatively low-level cognitive processes like speech style. Second, independently manipulating an individual’s self-concept and external factors allows examination of the relative contributions of identity and environment to speech style. If styleswitching occurs primarily in response to external factors (e.g., accommodation), we would expect an interlocutor (and the goals one may have with regard to that interlocutor) to play a decisive role in determining the occurrence and extent of styleswitching. On the other hand, if some aspects of style are linked in a stable way to aspects of one’s identity, we may observe that manipulating an individual’s self-concept can influence some aspects of their speech, irrespective of their interlocutors.

To explore these questions, we tested whether priming one of a biracial Black/White individual’s racial identities influences ordinary, spontaneous speech. In an earlier study (Gaither et al., 2013) self-identified biracial Black/White individuals living in the greater Boston area were recruited for an in-lab videotaped social interaction study with either a Black or White interaction partner where they discussed affirmative action. Before this interaction, participants were randomly assigned to write for 7 min about the racial identity of one of their parents: either their White parent or their Black parent (see Chiao et al., 2006). Gaither et al. (2013) showed this prime significantly affected participants’ levels of racial identification in accordance with the racial prime: biracial Black-primed participants identified more with other Black people while White-primed participants identified more with other White people. Additionally, this racial prime altered social behavior: participants primed with the same racial identity as that of their interaction partner (i.e., White prime and White interaction partner) had significantly more positive interactions (i.e., lower levels of anxiety and increased eye contact) than participants who had the opposite racial identity primed (i.e., White prime and Black interaction partner). Considered together these findings critically demonstrate that racial identity priming influenced the self-concept of the biracial participants, influencing in turn their explicit and implicit social behavior. To determine whether this shift also influences linguistic behavior (i.e., induces styleswitching), the conversations between these participants and their interaction partners were analyzed in the present study.

As mentioned before, dialects or ‘varieties’ of languages can differ at all levels of linguistic structure, from the principles that govern sentence formation down to the specifics of how various sounds are articulated. While regional variation is perhaps the most generally recognized (and oldest studied) form of linguistic variation, linguistic features are also known to co-vary with racial and ethnic identity (e.g., Boberg, 2004; Slomanson and Newman, 2004; Szakay, 2008; Newman and Wu, 2011; Benor, 2012). These features are not biologically determined (just as regional variants are not biologically determined) rather they represent particular linguistic principles learned (implicitly or explicitly) by particular communities of individuals. Given these correlations, listeners often use linguistic features to make inferences about a speaker’s social identity. While some inferences may relate to social stereotypes (that is, cultural values may become associated with particular features, e.g., Johnson and Buttny, 1982; Koch et al., 2001, see Baugh, 2003 and Cavanaugh, 2005 for reviews) other inferences may simply relate to the fine-grained statistical co-variation of linguistic features and identity (e.g., Labov et al., 2006; Warren et al., 2007; Smith et al., 2010; Newman and Wu, 2011).

Relatedly, past work has also shown that listeners are extremely accurate in identifying Black versus White speakers (for a review, see Thomas and Reaser, 2004) and research has suggested that there are phonetic characteristics that listeners associate with African American speech (e.g., Walton and Orlikoff, 1994; Purnell et al., 1999). Therefore, we hypothesized that participants primed with their Black identity would sound more ‘Black’ and those primed with their White identity would sound more ‘White’ to outside listeners. In Studies 1a,b, naive coders were recruited to assess whether identity priming does in fact shape biracial individuals’ speech. Inspired by our findings, we also sought to determine the dimensions along which identity priming can shape biracial Black/White individuals’ style. Therefore, in Studies 2a–c we investigated whether identity priming influences the degree to which such individuals utilize common linguistic features of African American English (AAE). Together, the goal of these studies was to shed light not only on the relationship between the cognitive construct of identity and language use but also how this identity can interact with the specifics of linguistic knowledge.

## Study 1a – Full Audio Coding

### Method

Audio clips were extracted from each of 56 interactions to include only the voice of the biracial participant. This previous study was university IRB approved and informed consent was obtained from all participants. No utterances of the confederate’s voice were extracted to ensure that only the voice of the biracial participant would be coded. Some audio could not be extracted either due to poor audio quality (*n* = 10) or an inability to cut out all occurrences of hearing the confederate’s voice (*n* = 2), resulting in a final sample of 44 3–4 min clips (12 Black-primed females, 12 White-primed females, 13 Black-primed males, seven White-primed males).

As our main dependent variable, four coders (two female; two White, one Asian, and one biracial Asian/White) rated each audio clip. These coders were research assistants who had no linguistic training and therefore represented how the average person would perceive these biracial speakers. While these research assistants were blind to condition and hypotheses, they were still knowledgeable about the various ways that Blacks and Whites are stereotyped—including the nature of their speech. Therefore, in line with how the average listener would hear different types of speech, coders rated each audio clip for how stereotypically Black to White participants sounded using a scale of 1 (*very Black*) to 7 (*very White*). In case a speaker’s position on affirmative action (the topic of the conversations) influenced perceived race, coders also rated the speaker’s perceived position on affirmative action 1 (*very opposed*) to 7 (*very in favor*). Lastly, to ensure that there were no differences in how anxious participants sounded (which could be equated with various prejudicial attributions such as being nervous or unprepared), coders also rated participants on how anxious to calm they sounded using a scale of 1 (*very anxious*) to 7 (*very calm*).

### Results

Across the four coders, one average rating was calculated for each rated item to create on rating per item (sounding Black: intraclass *r*= 0.63; sounding in favor of affirmative action: intraclass *r* = 0.81; sounding anxious: intraclass *r* = 0.75). As expected, Black-primed participants were rated as sounding significantly more Black (*M* = 3.26, SD = 0.73) than White-primed participants (*M*= 3.78, SD = 0.73), *t*(42) = 2.33, *p*= 0.025, *r*= 0.34. Black-primed participants were also rated as sounding significantly more in favor of affirmative action (*M* = 4.44, SD = 0.90) than White-primed participants (*M* = 3.95, SD = 0.61), *t*(42) = 2.05, *p*= 0.046, *r*= 0.30. Furthermore, ratings of sounding more White were also found to be positively correlated with sounding more opposed to affirmative action, *r* = 0.54, *p* < 0.01. To examine whether the biracial individuals’ stance on affirmative action may have influenced coders’ judgments as to whether the speaker sounded more Black or White, the coder’s ratings of sounding more in favor of affirmative action were included a as a covariate in a subsequent analysis. When doing so, the perceived group differences of Black-primed participants sounding more Black and White-primed participants as sounding more White was no longer statistically significant, *F*(1,41) = 2.04, *p* = 0.16.

**FIGURE 1:**
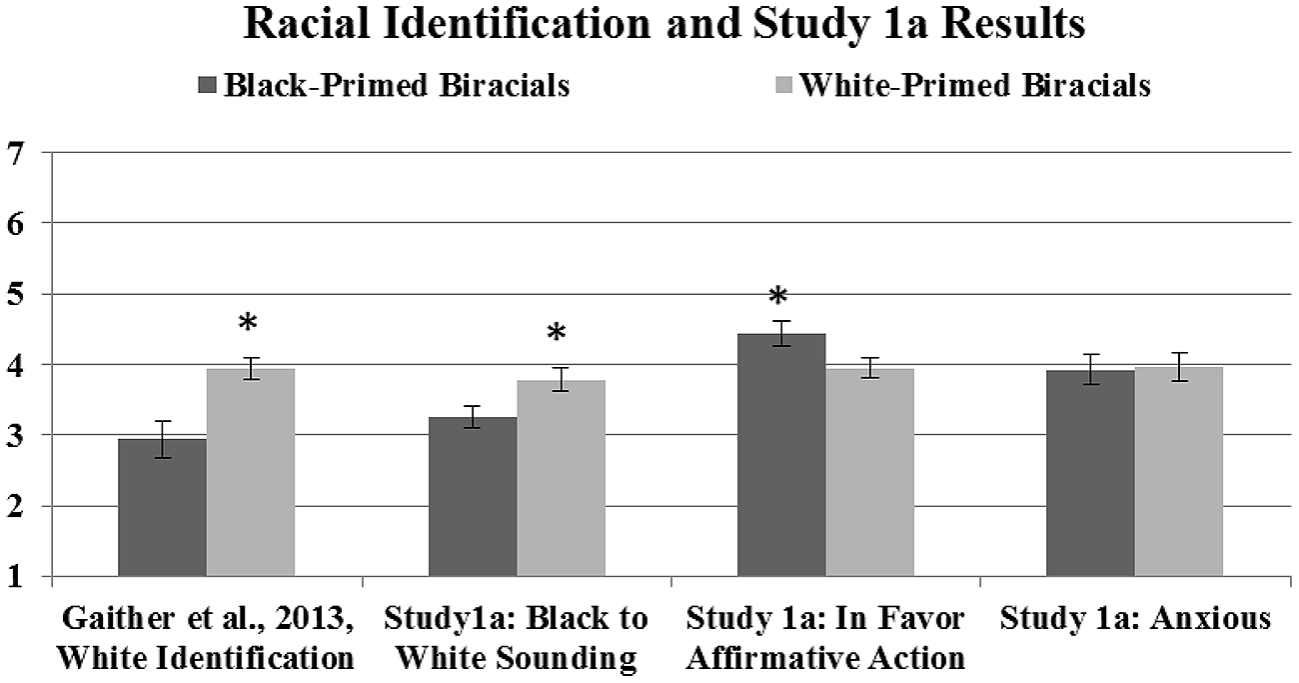
These means show the original self-reported racial identification of Black- and White-primed biracial participants from Gaither et al. (2013) in addition to the ratings from Study 1a. Lower numbers reflect identifying with *or* sounding more Black; higher numbers reflect identifying with or sounding more White and more in favor of affirmative action and anxious sounding; error bars represent SE; ^∗^denotes significant differences between priming groups.

There was no significant difference on sounding more anxious between Black-primed (*M* = 3.93, SD = 1.04) and White-primed participants (*M* = 3.97, SD = 0.89), *t*(42) = 0.15, *p*= 0.88 [see **Figure 1** showing the original Gaither et al. (2013) study racial identification results and these results]. Lastly, perceived phenotypicality ratings (degree of Black facial characteristics; see Maddox, 2004) of the biracial participants that were collected in the original study did not affect these outcomes. Therefore, appearing more physically Black did not affect how Black participants sounded.

Crucially, a 2 (primed identity: Black, White) × 2 (race of partner: Black, White) ANOVA revealed no interaction between identity prime and the race of one’s interaction partner on sounding more White to Black, *F*(1,40) = 0.15, *p* = 0.70. This suggests that the racial identity prime was strong enough to affect verbal behavior for biracial Black/White individuals irrespective of the race of their interlocutor, suggesting for the first time that styleswitching does not solely occur based on one’s interlocutor (or one’s goals with respect to the interlocutor). However, participants in this previous study were all racially primed before the interaction, meaning we still do not know if the race of one’s interaction partner would affect biracial speech when a biracial person’s identity is not explicitly primed. Therefore, future work should further explore the effects of interlocutors on biracial speech.

In sum, this study demonstrates that racial identity priming not only affects social behavior, but it also influences how Black or White a biracial individual speaks. However, despite our efforts at control, it is possible that coders may have relied on perceived positions on affirmative action when making their judgments of how Black/White they sounded. Therefore, in an effort to eliminate this possibility, Study 1b recoded these clips after eliminating all affirmative action related content.

## Study 1b – Thin Slice Audio Coding

### Method

Using a thin slicing approach (see Ambady et al., 2006 for similar methods), the same audio clips from Study 1a were shortened to 10–20 s segments that excluded all specific mention of affirmative action or other minority related material to ensure that the content of the audio clips would not be affecting the ratings in this second study^1^. Four new coders (three female; three White, one Black) that again had no linguistic training and were blind to condition and hypotheses rated each participant’s thin slice audio clip on the following dimensions: (1) how stereotypically Black to White participants sounded using a scale of 1 (*very Black*) to 7 (*very White*); (2) how uninformed to informed (i.e., intelligent) participants sounded on a scale of 1 (*very uninformed*) to 7 (*very informed*); and (3) how unsure to confident participants sounded on a scale of 1 (*very unsure*) to 7 (*very confident*). These last two ratings were used to explore past findings stating that voices from certain stereotyped groups tend to evoke prejudices associated with those groups. More specifically, past work has shown that both Black and White listeners perceive Black speakers less favorably than White speakers on traits including intelligence, confidence and ambition (e.g., Johnson and Buttny, 1982; Koch et al., 2001). Additionally, to control for affect, coders were also asked to rate how positive the speakers sounded using a 7-point scale 1 (*very negative*) to 7 (*very positive*). Lastly, coders were asked to list what they thought the person was talking about in order to ensure that listeners could not infer that the participants were speaking about minority-related issues.

### Results

**FIGURE 2:**
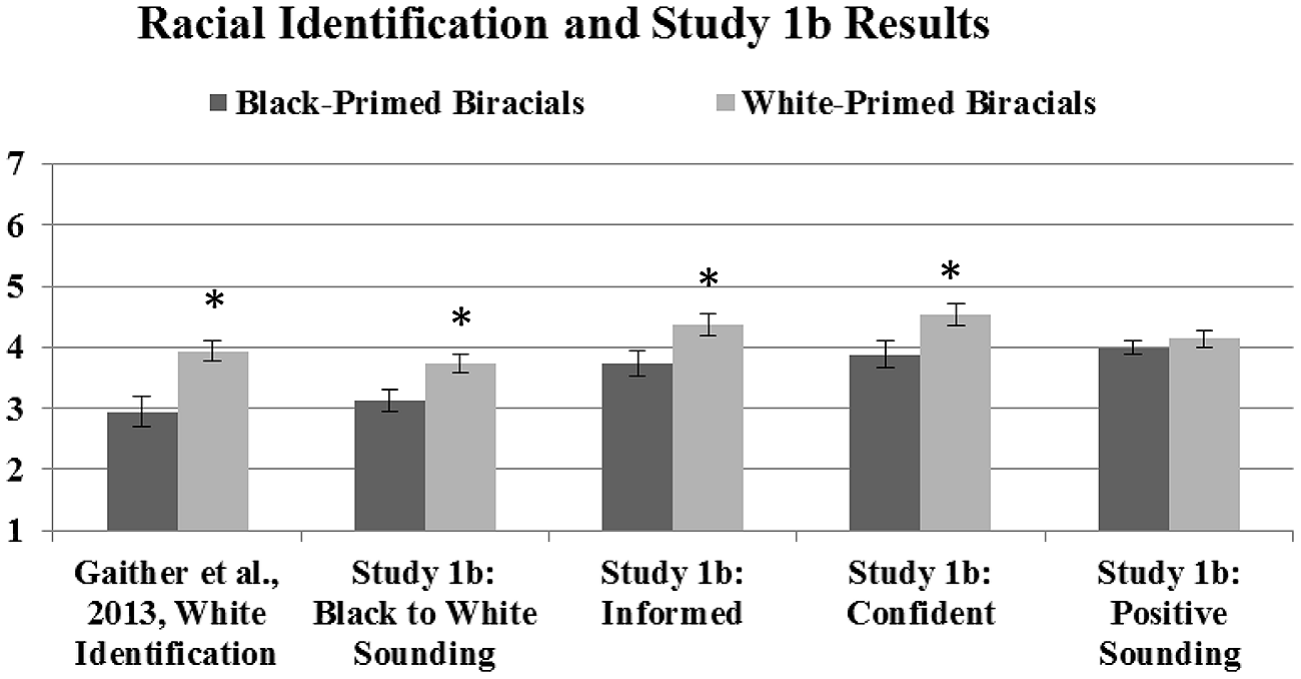
These means show the original self-reported racial identification of Black- and White-primed biracial participants from Gaither et al. (2013) in addition to Study 1b. Lower numbers reflect identifying with *or* sounding more Black; higher numbers reflect identifying with or sounding more White and more informed, more confident, and more positive sounding; error bars represent SE; ^∗^denotes significant differences between priming groups.

It is possible that a Black individual may perceive Black sounding speech differently than non-Black listeners, and since there were no Black coders in Studies 1a,b provided an opportunity to explore this possibility. However, we found high reliability across all coders regardless of their racial background, suggesting coder race was not a factor at least under the parameters of the present study. Therefore, an average rating was calculated for each rated item (sounding Black: intraclass *r*= 0.63; sounding informed: intraclass *r* = 0.72; sounding confident: intraclass *r* = 0.70; sounding positive intraclass *r* = 0.55). As in Study 1a, Black-primed participants were rated as sounding significantly more Black (*M* = 3.14, SD = 0.90) than White-primed participants (*M*= 3.73, SD = 0.69), *t*(38) = 2.29, *p*= 0.028, *r*= 0.35. Black-primed participants were also rated as sounding significantly less informed (*M* = 3.73, SD = 1.08) than White-primed participants (*M* = 4.36, SD = 0.80), *t*(38) = 2.10, *p*= 0.042, *r*= 0.32 and significantly less confident (*M* = 3.88, SD = 1.12) than White-primed participants (*M* = 4.53, SD = 0.80), *t*(38) = 2.09, *p*= 0.043, *r*= 0.32. There were no differences between Black-primed (*M* = 3.92, SD = 0.57) and White-primed participants (*M* = 4.14, SD = 0.57) on how positive they sounded, ruling out affect as a contributing factor in speech perception, *t*(38) = 1.22, *p*= 0.23. Furthermore, coders also did not list that any of the speakers were talking about any issues related to affirmative action. Lastly, as in Study 1a, neither phenotypicality nor the race of participants’ interaction partners affected these results [see **Figure 2** showing the original Gaither et al. (2013) study racial identification results and these results].

In sum, this study replicates findings from Study 1a in that racially priming biracial Black/White individuals significantly affects how they vocally sound to outside listeners—a fact that is not contingent upon the content of the speech or the racial background of the interlocutor. These results, combined with those from Study 1a, indicate that internal identity primes can manifest in speech, extending biracial identity flexibility research for the first time to verbal behavior. They also demonstrate that external factors such as interlocutors are not the only force that drives styleswitching behavior (e.g., Hartsuiker et al., 2004; Pickering and Garrod, 2004). This is not to say that interlocutors have no effect on one’s speech, a fact that has been clearly demonstrated in many previous studies. We will return to this issue in the Section “General Discussion.”

Having shown that racial priming influences the speech of biracial individuals, in Studies 2a–c, we conducted a linguistic investigation to determine if there are specific grammatical structures that differed between the White- and Black-primed participants. Dialects differ from each other at all levels of linguistic structure: the syntactic forms of sentences, the particular combinations of affixes used to form words, the particular sound patterns found within words, and the basic phonetic properties of the speech signal itself. The goal of these studies was to examine if certain linguistic properties may be involved in the styleswitching for biracial Black/White individuals. Since our participants were biracial Black/White, we contrasted the use of features commonly found in AAE (typically spoken by Black individuals, though by no means exclusively) and ‘General’ American English (GAE; a catchall term we use here as a proxy for the varieties of English most commonly spoken by White Americans). Study 2a investigated whether the speech of the Black- and White-primed participants differed in their syntactic and morphological properties, that is, features relating to the order of words in a sentence and the use of prefixes and suffixes to encode grammatical features. For example, the absence of the copula (e.g., ‘he crazy’) and the use of aspectual markers such as *bin* (e.g., ‘he bin working’) are common in AAE but not GAE and could serve as markers of Black identity (e.g., Rickford and McNair-Knox, 1994). In Study 2b, we investigated whether the two groups exhibited any differences in their use of a set of phoneme-level phonological patterns. Lastly, in Study 2c we examined whether the Black- and White-primed participants differed in their use of a number of handful low-level phonetic properties (e.g., pitch). To anticipate the results, we found no differences in the use of these features.

## Study 2a – Investigating Morpho-Syntactic Features

### Method

Transcripts of the thin-sliced audio clips from Study 1b were prepared by four transcribers naive to the purpose of the study. Each audio clip was transcribed word-for-word and false starts, disfluencies, filled pauses, and salient unfilled pauses were also included. As an example, the transcript for Participant 73 was as follows: “and, uhhhh, (pause) I haven’t really thought that much about it because it’s never really directly affected my life….” Standard orthography was used for all words (e.g., *running*, not *running*).

The written transcripts afforded us the opportunity to directly investigate whether there were differences in the syntactic or morphological patterns of the groups’ speech. By using transcripts, we isolated syntactic and morphological information while excluding phonological and phonetic factors from consideration. Ten coders who were blind to the purpose of the study were recruited to read and rate the transcripts individually using the same scales as Study 1b.

### Results

An average rating was calculated for each coded item and no significant differences by racial prime were found for (1) sounding more Black, (2) more informed, or (3) more confident (all *t*s < 0.54, all *p*s > 0.59). Therefore, we can infer that the differences perceived by the coders in Studies 1a,b are driven primarily by phonological properties and not the syntactic, morphological, or semantic content of the thin-slices. That is, these results suggest that the phonological and phonetic properties of the biracial participants’ speech are the dominant dimensions of styleswitching in these individuals.

## Study 2b – Phoneme-Level Features

### Method

The purpose of this study was to determine the extent to which the styleswitching of the Black- and White-primed groups involved categorical phonological patterns. Varieties of the same language frequently differ in which sounds appear in different environments. For example, American English speakers typically pronounce /t/ as a tap—[ɾ] , a rapidly articulated voiced consonant that sounds similar to [d]—when it appears between two vowels (compare the /t/ of *note* [noƱt] to that of *notable* [noƱɾәbәl] or *pity* [pIɾi]). In contrast, speakers of the Cockney variety of British English frequently pronounce intervocalic /t/ with a glottal stop—[Ɂ], the voiceless stop in the middle of *uh–oh*—such as in the word *pity* [pIɾi]. For the present study, four phonological patterns were identified that are not exclusive to AAE but are less common in GAE, particularly the variety spoken in the Boston area. These patterns were: interdental fricative substitution (e.g., GAE/AAE: *these* [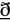
iz]/[diz], *brother* [brʌ
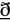

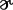
]/[brΛvә]), ‘g-dropping’ (e.g., *running* [rʌnɱ]/[rΛnIn]), final cluster reduction (e.g., desk [dεsk]/[dεs]); and final /l/- and /r/-deletion (e.g., *sore* [sɔr]/[sɔ]; *all* [ɔl]/[ɔ])^2^. Although cluster reduction may affect all word-final clusters, we excluded: (1) clusters ending in /t/ and /d/ (e.g., fast, bend) since these sounds are fr equently deleted in all varieties of American English (e.g., Patrick, 1999) and the size of our sample didn’t allow us to quantitatively distinguish between varieties; and (2) clusters ending in /s/ and /z/ since these clusters are rather unlikely to be reduced.

Two trained linguists blind to participant priming condition (Calvin L. Gidney and Ariel M. Cohen-Goldberg) conducted the present analysis. The first step was to identify all words in the thin-sliced transcripts that could possibly undergo the phonological rules listed above. For example, ‘g-dropping’ can only be observed in words that contain a word-final/ŋ/. The coders then compared their ratings and attempted to resolve any disagreements by repeated review of the tokens. In total, 339 words were identified that could possibly undergo one of the four phonological patterns described above, an average of 8.4 words per participant.

### Results

The coders initially disagreed on the coding of 17 of the 339 cases and were able to resolve all but four of the disagreements. We report the data with these remaining cases excluded. Overall, 96% of the tokens were coded as having a GAE pronunciation. AAE pronunciations were observed in 4/28 potential cases of ‘g-dropping,’ 8/116 potential cases of /l,r/-deletion, 1/38 potential cases of final cluster reduction, and 0/153 potential cases of interdental fricative substitution. In addition, two cases of unstressed syllable deletion and two cases of monophthongization were incidentally observed. These AAE features were observed in only nine of the 40 participants and the use of AAE phonological features did not differ across the two priming groups: five of the participants (nine observed AAE features) were in the White-prime condition while four participants (five observed AAE features) were in the Black-primed condition. Lastly, seven of the nine participants who produced AAE pronunciations used only one pattern; the other two used two patterns. On average, participants in the White prime condition produced more slightly more segments with the AAE variant (*M* = 4.3%, SD = 10%) than participants in the Black prime condition (*M* = 3.3%, SD = 7%) of the time but this difference was not significant *t*(38) = 0.36; *p*= 72; *d* = 0.12. These results suggest that the speech of Black- and White-primed individuals was not primarily distinguished by the four discrete sound-level properties examined here.

## Study 2c – Phonetic Features

Fundamentally, speech is a physical act involving the coordination of many different components of the vocal tract. Most speech sounds used in the world’s languages begin with the controlled exhalation of air from the lungs. During this process, the vocal folds rapidly open and close, adding periodic energy—voicing—to the airstream. Voicing plays an important role in many speech sounds and the frequency of vocal fold vibration determines the pitch of one’s voice. Speakers then move the tongue, lips, and velum in a highly coordinated fashion to further shape the airstream, producing individual speech sounds. The specific ways that sounds are physically articulated differ across languages and dialects/varieties and thus form part of a speaker’s knowledge of his or her language. In this study we examined five phonetic features that have previously been described as differing to some degree between AAE and GAE: jitter, shimmer, harmonics-to-noise ratio (HNR), utterance-wide pitch, and the degree of monophthongization in the vowel .

Jitter and shimmer quantify the magnitude of the variation in the timing and intensity (frequency and amplitude), respectively, of consecutive vocal fold closures while HNR quantifies the amount of noise in the speech signal. Walton and Orlikoff (1994) reported that speakers of AAE tend to exhibit more jitter and shimmer than speakers of GAE while Purnell et al. (1999) reported that AAE speakers tend to exhibit reduced HNR relative to GAE speakers. Other studies have reported that AAE and GAE speakers may differ in the extent to which their pitch (the highness or lowness of one’s voice, defined as the fundamental frequency of vocal fold vibration) may vary across an utterance. Loman (1975) and Jun and Foreman (1996) report that AAE speakers tend to exhibit greater changes in pitch across utterances than GAE speakers.

Finally, AAE is known to exhibit a greater degree of monophthongization than many (but not all) varieties of English spoken by White Americans. Vowels can generally be classified as monophthongs—vowels such as/i/ (*feet*), /I/ (*fit*), or /u/ (*food*) that involve a static placement of the lips and tongue—and diphthongs, which are vowels such as a /i/ (*time*),/aƱ/ (*pout*), and /ƆI/ (*toy*) that involve a trajectory of the tongue and possible change in lip rounding. Monophthongization is the tendency in Southern U.S. and African American dialects for diphthongs to be pronounced as monophthongs (e.g., pronouncing the /aI/ in *time* as [a:]). AAE speakers are more likely to exhibit monophthongized vowels than GAE speakers (Fasold and Wolfram, 1970). Interestingly Hay et al. (1999) analyzed recordings of The Oprah Winfrey Show and found that Winfrey was significantly more likely to monophthongize /aI/ when introducing an upcoming African American guest than a non-African American guest. The fact that monophthongization may occur when the interlocutor is not present suggests that it may be a good candidate for the sort of styleswitching being investigated in the present study^2^.

### Method

#### Jitter, Shimmer, HNR, and Pitch

The same thin-slice audio clips from Study 1b were used and all phonetic measurements were conducted using the Praat software package (Boersma and Weenink, 2013). The speech data were prepared for analysis by first extracting pitch and harmonicity data for the entirety of each clip. Pitch was measured using a forward cross-correlational method for the jitter and shimmer analyses and an autocorrelational method (Boersma, 1993) for the pitch analysis. Harmonicity data for the HNR analyses were measured using a forward cross-correlational method. The cross-correlational and autocorrelational techniques are recommended for voice and intonation analyses, respectively. Phonetic measurements were performed in two ways. In the first analysis phonetic measurements were taken over the entire clip, producing a single value for jitter, shimmer, etc. for each subject. *T*-tests were then used to determine whether the means of these measurements differed by priming condition (Black Prime, White Prime). Subsequently, a more fine-grained analysis was conducted. First, all of the vowels in each clip were automatically identified and transcribed using the Penn Phonetics Lab Forced Aligner (Yuan and Liberman, 2008). The phonetic measurements were then performed for each vowel, giving multiple values for each subject. These fine-grained data were then analyzed using linear mixed-effects regressions (described below) which provided greater power and allowed us to control for a number of important nuisance variables.

#### Monophthongization

The degree to which a vowel is articulated as a monophthong or diphthong can be assessed acoustically by examining the first and second formants. Formants are the frequencies of the speech signal that have the highest energy—the lowest such spectral peak is called the first formant (‘F1’) while the next lowest is called the second formant (‘F2’). For this analysis, we examined the vowel /aI/ obtained from tokens of the first person pronoun *I*. In the diphthong /aI/, F1 generally falls in frequency over the course of the vowel while F2 generally rises, consistent with the tongue moving higher and farther forward, respectively, over the course of vowel articulation. In contrast, F1 and F2 remain relatively unchanged in frequency the course of the monopthongized counterpart of this vowel, /a/. Since a vowel’s formant structure is influenced by its neighboring sounds, we sought to standardize the measurements by measuring the same word in each participant. In the end, all measurements were made from the first person pronoun *I* since this word was uttered by nearly all of the participants.

### Results

#### Jitter, Shimmer, HNR, and Pitch

*T*-tests of clip-wide values revealed no significant differences across the Black- and White-primed participants: the average jitter (measured as Relative Average Perturbation) for Black-primed participants (*M* = 0.016, SD = 0.01) did not differ from White-primed participants (*M* = 0.017, SD = 0.01), *t*(36) = 0.24, *p* = 0.81; *d* = -0.10; the average shimmer (local, db) for Black-primed (*M* = 1.63, SD = 0.11) and White-primed participants (*M* = 1.62, SD = 0.11) did not differ, *t*(36) = 0.291, *p* = 0.77; *d*= 0.09; and the average HNR for Black-primed (*M* = 5.57, SD = 1.24) and White-primed participants (*M* = 5.73, SD = 1.40) also did not differ, *t*(36) = 0.37, *p* = 0.72; *d* = -0.12. Lastly, there were no differences in the degree of pitch variation (average SD) between Black-primed (*M* = 35.39, SD = 14.13) and White-primed participants (*M* = 41.30, SD = 27.53), *t*(36) = 0.87, *p* = 0.39; *d* = -0.27, but a marginally significant difference was found in minimum pitch, with Black-primed participants (*M* = 83.35, SD = 20.39) having a lower minimum pitch than White-primed participants (*M* = 100.17, SD = 35.69), *t*(36) = 1.81, *p* = 0.08; *d* = -0.58.

The data were then analyzed on a token-by-token basis using linear mixed-effects modeling, a form of multiple regression where random effects may be entered into the model along with fixed effects. This technique allowed us to account for the fact that the participants’ speech varied in a number of ways (e.g., number of tokens, distribution and duration of vowels) and were non-independent in that each participant produced multiple tokens. For these analyses, each dependent variable was modeled as a function of a set of six fixed effects: Vowel (as coded by the Penn Aligner, Baseline = ‘AA’), Token Ordinal Position within the thin-slice clip, Vowel Stress (Unstressed = -1, Stressed = +1), Vowel Duration (measured in milliseconds), Speaker Sex (Male = -1, Female = +1), and Speaker Priming Condition (White Prime = -1, Black Prime = +1). In addition, the maximal random effects structure that would reliably converge was included in the model. Random intercepts for Participant and Word and random slopes for stress and duration (grouped by participant) were included in each model. Under this approach, any significant result is significant by both participants and items. Outliers were removed before analysis by fitting the model to the data and removing any data points whose standardized residual was greater than ±2.5 (Baayen, 2008).

The results of the six analyses are presented in **Table 1**, which reports the beta weight estimate, SE of the estimate, and *t*-value for each fixed effect. The number of tokens varies across analyses since the different measurements could not be made on all tokens (e.g., failure to estimate a token’s fundamental frequency). Generally speaking, predictors with *t*-values ≥ 2 are significant in models with large datasets such as the ones reported here (in the table predictors with *t*-values ≥ 2 are highlighted; significant results for vowel identity are not highlighted for clarity). Although all of the nuisance variables were significant in at least one model, Priming Condition was never significant. This provides additional support that that the identity prime manipulation did not significantly influence the speakers’ jitter, shimmer, HNR, or pitch.

**Table 1:** Results of linear mixed-effects analyses for six phonetic properties.

	Log jitter (*n* = 1186)			Shimmer (*n* = 1146)			HNR (*n* = 1474)			Log pitch min (*n* = 1387)			Log pitch max (*n* = 1390)			Log pitch SD (*n* = 1303)		
Predictor	β	SE(β)	*t*	β	SE(β)	*t*	β	SE(β)	*t*	β	SE(β)	*t*	β	SE(β)	*t*	β	SE(β)	*t*
(Intercept)	-5.38	0.21	-25.1	1.40	0.12	11.3	4.67	0.70	6.7	4.98	0.06	82.3	5.03	0.06	85.0	0.17	0.21	0.8
Vowel-AE	0.85	0.24	3.6	0.15	0.14	1.1	-1.42	0.72	-2.0	0.01	0.05	0.3	-0.06	0.05	-1.1	-0.03	0.22	-0.2
Vowel-AH	0.75	0.21	3.5	0.21	0.13	1.7	0.11	0.63	0.2	0.03	0.04	0.6	-0.02	0.05	-0.6	-0.07	0.20	-0.3
Vowel-AO	0.46	0.28	1.7	0.06	0.16	0.4	1.05	0.83	1.3	-0.05	0.06	-0.8	-0.03	0.06	-0.5	0.30	0.26	1.1
Vowel-AW	0.69	0.35	1.9	0.10	0.21	0.5	-0.86	1.14	-0.8	0.01	0.07	0.1	-0.07	0.08	-0.9	0.02	0.35	0.1
Vowel-AY	0.68	0.24	2.8	0.15	0.14	1.0	-1.34	0.73	-1.8	-0.04	0.05	-0.7	-0.07	0.05	-1.3	-0.14	0.22	-0.6
Vowel-EH	0.54	0.22	2.5	0.14	0.13	1.1	-0.31	0.66	-0.5	0.00	0.05	0.1	-0.02	0.05	-0.4	-0.04	0.21	-0.2
Vowel-ER	0.56	0.25	2.3	0.06	0.15	0.4	-0.26	0.73	-0.4	0.00	0.05	-0.1	0.01	0.05	0.1	0.09	0.24	0.4
Vowel-EY	0.82	0.25	3.3	0.13	0.15	0.9	1.60	0.74	2.2	-0.01	0.05	-0.3	-0.01	0.05	-0.2	0.12	0.24	0.5
Vowel-IH	0.95	0.22	4.4	0.32	0.13	2.5	0.64	0.64	1.0	0.04	0.05	0.9	0.04	0.05	1.0	0.49	0.21	2.4
Vowel-IY	1.00	0.22	4.6	0.28	0.13	2.2	1.94	0.65	3.0	-0.01	0.05	-0.2	-0.02	0.05	-0.4	0.21	0.21	1.0
Vowel-OW	0.45	0.26	1.8	0.06	0.15	0.4	0.32	0.77	0.4	0.01	0.05	0.2	0.00	0.06	0.0	0.08	0.24	0.3
Vowel-UH	0.86	0.31	2.8	0.17	0.19	0.9	1.63	0.92	1.8	0.07	0.07	1.1	0.07	0.07	1.1	0.32	0.30	1.1
Vowel-UW	0.96	0.27	3.6	0.02	0.16	0.1	2.90	0.77	3.8	0.01	0.05	0.2	-0.01	0.06	-0.1	0.26	0.25	1.0
Order	0.00	0.00	-1.0	0.00	0.00	0.3	-**0.01**	**0.01**	-**2.3**	**0.00**	**0.00**	-**6.9**	**0.00**	**0.00**	-**6.1**	0.00	0.00	-1.3
Stress-stressed	-0.10	0.05	-1.9	0.03	0.03	1.0	-0.07	0.15	-0.5	**0.03**	**0.01**	**2.0**	0.03	0.01	1.8	0.01	0.05	0.2
Duration	-0.10	0.42	-0.2	-0.23	0.25	-0.9	**6.79**	**1.34**	**5.1**	-**0.46**	**0.10**	-**4.5**	**0.26**	**0.11**	**2.5**	**5.24**	**0.62**	**8.4**
Sex-female	-0.10	0.08	-1.3	0.00	0.02	0.0	0.48	0.31	1.6	**0.21**	**0.04**	**4.7**	**0.18**	**0.04**	**4.7**	**0.21**	**0.08**	**2.5**
Condition-black	-0.03	0.07	-0.5	0.00	0.02	-0.2	0.35	0.30	1.2	-0.05	0.04	-1.1	-0.05	0.04	-1.4	0.01	0.08	0.1

*Coefficient estimates (β), SE (β), and t-values are reported for all fixed-effect predictors, as are the number of tokens analyzed in each regression. Predictors (other than Vowel predictors) with t-values ≥ 2 (indicating significance) are highlighted*.

#### Monophthongization

Measurements were obtained from the token of *I* judged to be acoustically clearest for each participant. Five participants did not produce the pronoun *I* during the thin slice and were excluded from these analyses; two additional participants were excluded due to excessive noise during the articulation of the pronoun (three Black-primed; four White-primed). Tokens were normalized for length by dividing each vowel into 10 equal time points. Formant measurements were made using Praat’s automatic formant tracker augmented with hand-specified parameters for number of formants and frequency ceiling. Monophthongization was measured by calculating the difference between F2 and F1 at each of the 10 time points and fitting a regression line to these differences. A positive slope of the regression line would indicate that the difference between F2 and F1 grew over time (consistent with articulation as a diphthong), a negative slope would indicate that the difference became smaller over time, and a slope near 0 would indicate no change over time (consistent with articulation as a monophthong). An independent samples *t*-tests revealed no difference in average slope between Black-primed (*M* = 34.29, SD = 28.47) and White-primed participants (*M* = 45.56, SD = 31.67), *t*(29) = 1.04, *p* = 0.31; *d* = -0.37.

## General Discussion

Our results demonstrate for the first time that the experimental manipulation of a social psychological variable (racial identification) leads to a real-time shift in speaking style. As demonstrated by Gaither et al. (2013), biracials are sensitive to these primes in guiding their behavior. In this paper, we show that this influence extends to their patterns of speech, with implications for how they may be perceived by others. Sounding black is sufficient to activate cultural stereotypes, potentially biasing subsequent evaluations (e.g., Johnson and Buttny, 1982; Koch et al., 2001). These results hold important theoretical and methodological implications for both the social psychological and sociolinguistic literatures. First, the demonstration that momentary shifts in social identity can be expressed through speech broadens our understanding of identity as a psychological phenomenon, indicating that intimate links exist between social and linguistic cognitive processes. Second, these results extend biracial identity flexibility research to language, highlighting another commonality between biracial and bicultural populations through social identification and language use. Lastly, our study suggests that language could potentially be used as an implicit index of social identification in laboratory experiments, complementing more traditional measures such as ratings.

Our results also enrich the sociolinguistic literature. First, they demonstrate that styleswitching can occur in response to the internal state of the individual, not simply in response to the individual’s environment. This suggests that at least some components of style are stably linked to aspects of a speaker’s identity and may manifest as those aspects become prominent. Second, our results suggest that social psychological techniques—identity priming in particular—may be a useful addition to the sociolinguist’s toolbox, allowing the research to independently manipulate speaker identity and context.

Individuals with multiracial identities face unique challenges in navigating the social landscape by adopting specific cognitive strategies that enable them to associate more with one racial identity as needed (e.g., Chiao et al., 2006; Bonam and Shih, 2009; Gaither et al., 2013). We believe that the racial priming utilized in this study is (at least temporarily) changing the internal or personal racial identification of the participant which in turn directly affects their verbal behavior (Gaither et al., 2013). We show that styleswitching for biracial individuals is more of a holistic phenomenon since it affects participants’ speech overall, not just the words they choose to use. Furthermore, this explicit prime causes the focus to be on one’s own identification rather than the group membership of the interlocutor. However, other work suggests that additional research is needed to investigate the situational factors that may prime styleswitching abilities (Fu et al., 2007). Future work should examine whether biracial individuals who are not explicitly primed naturally styleswitch based on the racial background of their interaction partner. Furthermore, this study included audio analysis only of biracial Black/White individuals—it is imperative to study other mixed-race populations (especially those who grew up speaking more than just variations of English) and other linguistic markers to investigate whether these findings apply more generally to the mixed-race demographic.

One particularly interesting finding is that although the styleswitching was apparent to listeners, the specific linguistic manifestations of this shift were less clear. This, however, has precedent in the literature. For example, Gaudio (1994) reported that listeners could reliably judge speakers’ sexual orientation based on short audio passages even though he was not able to instrumentally find reliable phonetic differences between gay and straight speakers (see also Moonwomon, 1985). And similar difficulties have been noted in identifying which features listeners use to distinguish Asian American (Newman and Wu, 2011) and Black speakers (Thomas and Reaser, 2004).

While it is always difficult to interpret null results, we believe a number of factors may have contributed to our failure to instrumentally find specific linguistic markers of Black/White identity. The first and most straightforward account is that participants utilized linguistic features to indicate identity that were simply not covered in our analysis. While we examined many of the prominent features that distinguish these varieties (and indeed, some that have been shown to specifically manifest in styleswitching), many features were not analyzed and these gaps should be filled in future studies. Second, the Black-primed participants exhibited many grammatical markers of GAE while still “sounding Black” to our coders. This suggests that there are possibly other characteristics of speech that listeners strongly associate with African American speech that have yet to be empirically documented (e.g., Walton and Orlikoff, 1994; Spears, 1998; Thomas and Reaser, 2004; see Munson, 2011 for a general discussion of the difficulties surrounding phonetic ‘parameterization’). Third, environmental factors may have played an important role in shaping the linguistic competence of our biracial speakers. While it is common for monoracial individuals to grow up in a household where both parents speak the same dialect, this likely was not the case for our participants. Our biracial participants may thus have been exposed to greater heterogeneity in their linguistic experience, causing their speech to incorporate subtle shifts in speech patterns that are more difficult to detect or label empirically. There may also be heterogeneity in the way speakers shift between Black and White speech—some speakers may adopt particular features and not others (Zwicky, 1997). Our analyses—and statistical tests—considered each specific feature in isolation. Given that the properties measured in the present analyses are rather small (typical Cohen’s *d*-values were ∼0.1), it is possible that our sample size was too small to reliably detect these differences. It is also likely that each feature contributes in a weighted fashion to the listener’s percept of the speaker’s linguistic identity, making analyses of individual features less likely to reliably distinguish between AAE and GAE speech. For example, while Newman and Wu (2011), identified a number of properties used by listeners to identify Asian American speakers, no feature was used by all speakers. Thus, listeners may utilize a mosaic of “separate pieces of individually weak evidence […] to yield a judgment” that has a high probability of being correct (Liberman, 2010, cited in Newman and Wu, 2011). Finally, it may be that a fundamental component of navigating a Black/White biracial identity in the U. S. is the maintenance of a subtle (rather than overt) blend of one’s dialects (Zwicky, 1997). We offer these possibilities as avenues for future research.

Overall, these results underscore the importance of examining the intersections between social identity and all forms of behavior—social and verbal—especially for biracial individuals who may exhibit different speaking strategies based on salient racial identities. Most importantly, this work emphasizes the need for social psychological and linguistic research to further define their methods to include biracial populations who do not seem to fit within the currently established methods and frameworks—frameworks that were constructed originally based on the study of monoracial populations. This is further support that the biracial population contradicts the traditional social construction of race but extends that contradiction to language for the first time. With the mixed-race population estimated to be over 25% of the total population within the next 40 years (with biracial Black/White individuals being the most commonly reported, U.S. Census, 2010), it is time to change our methods and frameworks to be more in line with our changing demographic.

## Author Contributions

SG and KM were responsible for the original research question and design. AG and CG were responsible for designing and implementing linguistic analyses. SG and AG completed all data analysis with guidance from KM and CG All authors agreed to be accountable for all aspects of this work and ensured its accuracy and integrity. All authors also significantly contributed to the writing and final approval of this manuscript.

## Conflict of Interest Statement

The authors declare that the research was conducted in the absence of any commercial or financial relationships that could be construed as a potential conflict of interest.

## Footnotes

1 Four audio clips were excluded from analysis in Study 1b because there was not a 10–20 s clip available that was free of race-related content, resulting in a final sample of 40 thin slice audio clips (11 Black-primed females, 12 White-primed females, 10 Black-primed males, seven White-primed males).

2 Although /r/-deletion is common in the varieties of English spoken in New England, we included it in our analysis since it is less common among younger speakers and speakers with more education (Irwin and Nagy, 2007), demographics that described the majority of our biracial participants. Further, /l/-deletion is uncommon in New England, making it potentially diagnostic of African American English (AAE).
